# The effects of magnesium supplementation on abnormal uterine bleeding, alopecia, quality of life, and acne in women with polycystic ovary syndrome: a randomized clinical trial

**DOI:** 10.1186/s12958-022-00982-7

**Published:** 2022-08-02

**Authors:** Mahsima Jaripur, Hatav Ghasemi-Tehrani, Gholamreza Askari, Mahsa Gholizadeh-Moghaddam, Cain C. T. Clark, Mohammad Hossein Rouhani

**Affiliations:** 1 Food Security Research Center and Department of Community Nutrition, School of Nutrition and Food Science, Isfahan University of Medical Sciences, Isfahan, Iran; 2 Infertility & IVF Fellowship, Department of Obstetrics and Gynecology, Isfahan University of Medical Sciences, Isfahan, Iran; 3 Centre for Intelligent Healthcare, Coventry University, Coventry, CV1 5FB UK

**Keywords:** Polycystic ovary syndrome, Magnesium, Acne, Quality of life, Alopecia, Abnormal uterine bleeding

## Abstract

**Background:**

Abnormal uterine bleeding (AUB), alopecia, low quality of life, and acne are considered as complications of polycystic ovary syndrome (PCOS). We hypothesized that magnesium supplementation would yield beneficial effects on PCOS related complications.

**Objective:**

To examine the effects of magnesium supplementation on AUB, alopecia, quality of life, and acne.

**Methods:**

In this parallel randomized clinical trial, we randomly assigned 64 women with PCOS to the magnesium group (*n *= 32) or placebo group (*n* = 32) for 10 weeks. AUB, alopecia, quality of life, and acne were assessed by the International Federation of Gynecology and Obstetrics criterion, the Sinclair Scale, the Health Survey Quality of Life Questionnaire, and the Global Acne Grading System, respectively. This randomized clinical trial was registered at IRCT.ir (IRCT20130903014551N9).

**Results:**

Magnesium supplementation significantly improved the components of quality of life including physical functioning (*p* = 0.011), role limitations due to physical health (*p* = 0.012), role limitations due to emotional problems (*p* < 0.001), energy/fatigue (*p* = 0.005), emotional wellbeing (*p* < 0.001), social functioning (*p* = 0.002), general health (*p* = 0.013), and total quality of life (*p* < 0.001), compared with placebo. No significant effect was observed on acne, alopecia, and AUB.

**Conclusion:**

Magnesium supplementation in women with PCOS had a significant positive effect on improving total quality of life.

**Trial registration:**

This randomized clinical trial was registered at IRCT.ir on 2020–10-18 (Registration Code: IRCT20130903014551N9).

**Supplementary Information:**

The online version contains supplementary material available at 10.1186/s12958-022-00982-7.

## Introduction

Polycystic ovary syndrome (PCOS) is characterized by several cysts and follicles in enlarged ovaries, and production of infertile eggs [1]. Genetic and environmental factors, including dietary intakes, are contributing factors of PCOS [2]. The World Health Organization estimates that 116 million women worldwide have PCOS; and its prevalence varies from 2 to 26%, globally [3]. Symptoms of PCOS include abnormal uterine bleeding (AUB) and signs of excess androgens secretion, such as acne, male pattern alopecia, and low quality of life [1].

AUB is prevalent among women with PCOS; indeed, evidence suggests that 50% of patients had oligomenorrhea and 20% had amenorrhea [4]. Androgenic alopecia is another complication of PCOS and it is a leading causes of hair loss in women [5], where, up to, 67% of women with PCOS suffer from androgenic alopecia [5]. One of the most common signs of androgen overload in PCOS is acne [6], which is an inflammatory disease of the hair follicles and apocrine glands that occurs in more than one-third of women with PCOS [7]. Researchers have shown that the quality of life in women with PCOS is lower than healthy subjects and even compared to those women with other gynecological diseases [8]. Infertility, menstrual irregularities, hirsutism, acne, hair loss, anxiety and depression are possible causes of low quality of life in PCOS [9].

Evidence suggests that magnesium deficiency may play an important role in women's health in several clinical conditions, including premenstrual syndrome, dysmenorrhea, and PCOS [10]. Women with PCOS have lower serum magnesium levels than healthy people [11]. Magnesium can help reduce menstrual pain and cramps [12], and is involved in the formation of proteins, cell growth, and division cell involved in hair. Therefore, it is posited that magnesium intake can improve hair loss in women [13]. Magnesium may also have beneficial effect on skin lesions and acne; for instance, previous studies have shown that magnesium improves collagen production in the skin, whilst low magnesium intake may cause inflammation [14]. Also, serum magnesium levels have been shown to be low in patients with acne [15], and co-supplementation of magnesium and myoinositol was reported to improve acne [16] Magnesium may have favorable effect on components of quality of life including depression [17, 18], where previous studies showed that magnesium supplementation improved depression in diabetic and non-diabetic patients [19, 20].

According to previous studies, we hypothesized that magnesium supplementation might elicit beneficial effects on complications of PCOS. Therefore, this study sought to evaluate the effect of magnesium supplementation on AUB, alopecia, quality of life, and acne in women with PCOS.

## Method

This study was carried out in the period of November 2020 to November 2021 in Isfahan, Iran. Subjects were included if they: 1) were aged 18 to 45 years old; 2) were diagnosed with PCOS according to the Rotterdam criteria [21]; 3) had no change in the dose of the medications or did not start taking a new medication during the previous 14 days; 4) were not in menopause; and 5) did not take vitamin and mineral supplements. Subjects who changed dose of medications or started taking new drugs were excluded. Also, we excluded patients who were pregnant or menopausal during the study.

To find the eligible participants, we screened the records of subjects who registered as PCOS patients in Shahid Beheshti Obstetrics and Gynecology Hospital, Isfahan, Iran. We called them to evaluate whether they had signs and symptoms of PCOS. Then women who reported signs and symptoms of PCOS were invited to run an assessment based on the Rotterdam criteria to ensure that they had PCOS. According to the Rotterdam criteria, subjects who had two of the following three symptoms were diagnosed as having PCOS: 1) anovulation or ovulatory dysfunction; 2) increased serum concentration of androgens; and 3) at least 12 follicles in each ovary known as polycystic ovaries on ultrasound [21]. Before including in the study, women were assessed for these criteria and then subjects who had two of the three symptoms were selected for the study. The International evidence-based guideline for the assessment and management of PCOS emphasizes where irregular menstrual cycles and hyperandrogenism are present, ultrasound is not necessary. Therefore, ultrasound was not performed for women with irregular menstrual cycles and hyperandrogenism[22] All subjects were outpatients referred to clinic of Shahid Beheshti Obstetrics and Gynecology Hospital, Isfahan, Iran. All patients enrolled in this study wanted to be pregnant. Shahid Beheshti Obstetrics and Gynecology Hospital focuses on infertility and women who want to be pregnant are referred to this center. Subjects referred to this center are categorized based on the main cause of infertility. We used records of subjects who could not be pregnant because of PCOS. We did not include admitted women.

To calculate required sample size, score of quality of life was considered as the main outcome variable. Based on the previous studies, we considered ∆ = 0.47 and S^2^ = 0.66 [23]. According to the following equation, in which α = 0.05 and β = 0.20 (the power of the study was 80%), the estimated minimum sample size in each group was 30:

*n* = 2 [(Z1-α / 2 + Z1-β) ^2^ × S^2^] / Δ^2^ = 2 [(1.96 + 0.85) ^2^ × (0.66) ^2^] / (0.47) ^2^ = 30.

Finally, 64 subjects (*n* = 32 in each group) were included in the study because of possible withdrawal. Participants were randomly allocated in a ratio of 1:1 to either magnesium supplement or placebo using a computer-generated randomization sequence. We did not use blocks in randomization. We assigned a code to each subject and entered the codes into SPSS. Then participants were randomly divided in to 2 groups by SPSS.

Randomization list and numbering of supplements containers were performed by staff who had no contribution in the intervention and assessment of the outcomes. Therefore, investigators who evaluated outcomes were blinded. All participants signed a written consent form prior to participation. This study was ethically approved by The Research Council and Ethical Committee of Isfahan University of Medical Sciences, Isfahan, Iran, (Code: IR.MUI.RESEARCH.REC.1399.406). This randomized clinical trial was registered at IRCT.ir (IRCT20130903014551N9).

### Intervention

Comprehensive information regarding the study were explained to participants. In the magnesium group, a 250 mg magnesium oxide tablet (Magni One® produced by DonyaDarou, Tehran, Iran) per day was administered for 10 weeks. In placebo group, we used a tablet that contained 5 mg starch and its color, appearance, smell, and taste were similar to the 250 mg magnesium oxide tablet. Participants were asked to consume tablets after breakfast. We used telephone calls and virtual networks to monitor use of supplements.

Women in both groups received a list of dietary recommendations, including: 1) limit consumption of refined or simple carbohydrates, such as white bread, white rice, sugar and sweets; 2) increase consumption of fresh vegetables; 3) use more mini-meals instead of big meals; 4) drink at least 8 glasses of fluid, especially water; 5) be cautious about your weight and avoid overeating; 6) consume leafy vegetables such as lettuce and cabbage instead of starchy vegetables such as potatoes; 7) increase consumption of fresh fruits and avoid using industrial and sugar sweetened fruit juices; 8) limit consuming salty foods, fast foods and high-fat dairy products; and 9) use healthy oils such as olive oil and canola oil and limit consuming saturated, partially saturated vegetable oil or animal fat.

### Evaluation of AUB

Based on the definitions provided by the International Federation of Gynecology and Obstetrics (FIGO), following criteria were considered as components of AUB [24, 25]:

1. Frequency of menses: The duration of the menstrual cycle is normally 24 to 38 days. Therefore, regular episodes of bleeding at intervals of ≤ 24 days or > 38 days were considered as abnormal.
2. Regularity of menses: Irregular menses was defined as shortest to longer cycle variation was ≥ 10 days.
3. Duration of menses: If the duration of menstrual bleeding was more than 8 days or less than 3 days in each period, it was considered as abnormal
4. Volume of monthly blood loss: If a woman's bleeding volume was between 5 to 80 ml in a period, it was considered normal and less than 5 ml or more than 80 ml was abnormal.

The number of AUB criteria in each subject was assessed at baseline and after 10 weeks of intervention.

### Evaluation of male pattern hair loss

Clinical manifestations of alopecia was assessed at the beginning and end of the study using the Sinclair Scale [26]. The validity and reliability of this method have been accepted in previous studies [27]. No manifestation of alopecia was defined as the first stage, alopecia in the center of the scalp was categorized as second stage, expanded alopecia in the center of scalp and hair loss in lateral area was considered as the third stage, in the fourth stage, a bald spot could be detectable on the anterior portion of the scalp, and finally, advanced alopecia was categorized as the fifth stage [28].

### Assessment of acne

To evaluate the severity of acne, we examined existence of acne according to Global Acne Grading System [29]. Validity and reliability of this method was acceptable in previous studies [30, 31]. In this scoring system, forehead, right cheek, left cheek, nose, chin, upper back, and chest were assessed. A factor was defined for each are: forehead = 2, right cheek = 2, left cheek = 2, nose = 1, chin = 1, chest and upper back = 3. We scored each type of lesion based on the severity: no lesions = 0, comedones = 1, papules = 2, pustules = 3 and nodules = 4. Local score for each area was calculated according to the following formula: Local score = Factor × Lesion score (0–4). The total score was calculated by summing local scores, and acne severity was defined as mild (score of 1–18), moderate (score of 19–30), severe (score of 31–38), and very severe (score of > 39) [29]. Clinical manifestations of acne was assessed at the beginning and end of the study.

### Assessment of Quality of Life

To assess the effect of magnesium on quality of life, we asked patients to complete the Health Survey Quality of Life Questionnaire (SF-36) before and after the intervention [32, 33]. The validity and reliability of this questionnaire was evaluated and the results were accepted [34, 35]. The SF-36 could assess eight scales: physical functioning (PF), role physical (RP), bodily pain (BP), general health (GH), vitality (VT), social functioning (SF), emotional role (ER), and mental health (MH) [36]. The total score was equal to the average of scores in each eight subscales. Higher scores were interpreted as higher quality of life [37].

### Assessment of physical activity

Physical activity of the participants was presented as metabolic equivalent per hour per day (MET.h.d). Each participant completed 5 one-day physical activity diaries during the study. Individuals were asked to report their activities such as walking, exercise, sleep, watching TV, housework, studying, bathing, and so on. The total metabolic equivalent was calculated by multiplying the frequency, duration, and intensity of each physical activity in 24 h.

### Dietary intake

To assess dietary intakes during the study, each participant was asked to complete 5 one-day food records, including 3 weekdays and 2 weekends. Nutrient content of the foods was calculated by Nutritionist IV based on the United States Department of Agriculture food composition database.

### Biochemical assessment

The serum level of magnesium was measured at baseline. A 5 ml blood sample was collected and serum was isolated. We measured magnesium by Atomic Absorption Spectrophotometry method.

### Socioeconomic status

To classify patients in terms of economic status, they were asked about the amount of family income and based on the amount of income, they were classified into three groups: 1) Poor economic status (for incomes less than three million ،Tomans per month), 2) Medium economic status (monthly income Between four to ten million Tomans) and 3) Good economic situation (for people whose average family income was above ten million Tomans per month). This division was based on living conditions in Iran and the income range of clients. The level of education of each person was asked, and they were divided into three groups: 1) under diploma, 2) Diploma, and 3) University education.

### Statistical Analysis

We ran an intention to treat (ITT) analysis by using the linear regression method in the current study [38]. The Kolmogorov–Smirnov test and visual inspection of Q-Q plots were applied to evaluate normal distribution, and no variables had a large deviation from normal distribution. The comparison of qualitative variables between the magnesium and placebo groups was conducted using the Chi-square test, whilst nominal and ordinal variables were reported as percentage. Within group comparison (baseline vs. endpoint) was performed using Paired T test analysis. Inter-groups comparisons were performed using Independent Student t-test for numerical variables. We adjusted the effect of the confounding variables (baseline serum magnesium, energy intake and baseline values) using analysis of covariance (ANCOVA). Scale variables were reported as mean ± standard deviation. All data analyses were conducted using SPSS version 21 statistical software, with an a priori alpha level of 0.05.

## Result

**Fig. 1:**
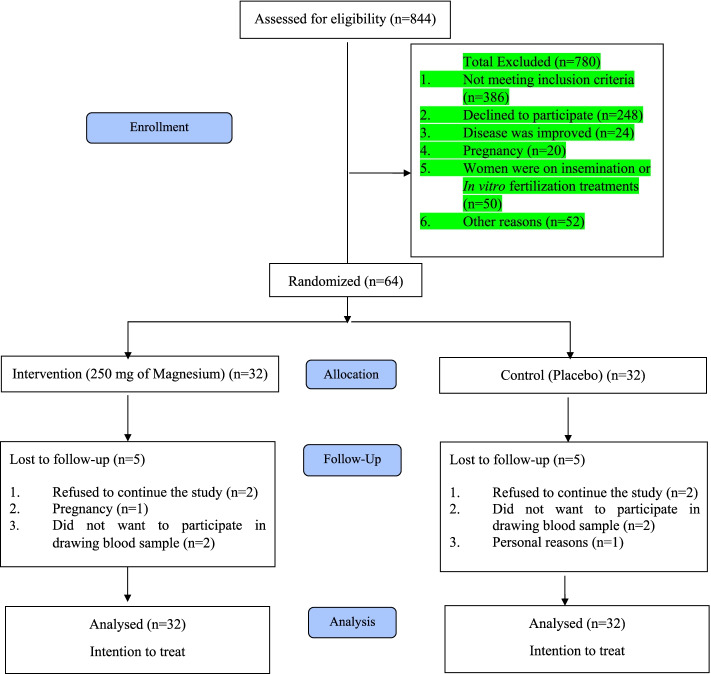
CONSORT study flow diagram

The process of patient recruitment is shown in Fig. 1. To find the eligible participants, we screened the records of outpatients referred to clinic of Shahid Beheshti Obstetrics and Gynecology Hospital, Isfahan, Iran. Initially, the records of subjects registered as PCOS patients were screened (*n* = 844). Then we called them and 780 patients were excluded because: 1) they did not meet the inclusion criteria (*n* = 376); 2) PCOS was treated (*n* = 24); 3) patients were on insemination in vitro fertilization treatments (*n* = 50); 4) they refused to participate in the study (*n* = 248); 5) they were pregnant (*n* = 20); or 6) other reasons (*n* = 52). Then women who reported signs and symptoms of PCOS were invited to run an assessment based on the Rotterdam criteria to ensure that they had PCOS [21]. Therefore, 64 patients were included in the study and they were randomly assigned into magnesium (*n *= 32) or placebo (*n* = 32). During the follow-up process, five patients in the magnesium group were lost to follow-up because they: 1) refused to continue the study (*n* = 2); 2) were pregnant (*n *= 1); or 3) did not want to participate in blood sampling (*n* = 2). Similarly, five subjects were lost to follow-up in the placebo group because they: 1) refused to continue the study (*n* = 2); 2) did not want to participate in blood sampling (*n* = 2); or 3) personal reasons (*n* = 1). Therefore, 54 patients completed the study. Nevertheless, data of 64 people (32 subjects in each group) were analyzed based on the ITT method.

Table 1 shows general characteristics of the participants. Results demonstrated that age (*p* = 0.615), height (*p* = 0.439), weight (*p* = 0.918), weight status (*p* = 0.987), BMI (*p* = 0.808), educational status (*p* = 0.382), economic status (*p* = 0.186), marital status (*p* = 0.306), and the level of physical activity (*p* = 0.733) were not different between two groups. Baseline serum magnesium was higher in the intervention group compared with placebo (p = 0.047). More data regarding physical activity are presented in the Supplementary File 1.

**Table 1:** General Characteristics of the participants

Variable	Magnesium (*n* = 32)	Placebo (*n* = 32)	P
Age (y)	31.69 ± 5.41^1^	32.44 ± 6.42	0.615
Weight (kg)	69.88 ± 14.36	70.22 ± 12.22	0.918
Height (m)	1.6 ± 0.07	1.62 ± 0.05	0.439
BMI (kg/m^2^)	26.89 ± 4.68	26.63 ± 4.06	0.808
Overweight/Obese (%)	68.5	68.7	0.987
Education (%)			
Did not complete high school	25	15.6	0.382
High school	46.9	40.6	
University degree	28.1	43.8	
Economic Status (%)			
Low	18.8	9.4	0.186
Medium	71.9	65.6	
High	9.4	25	
Married (%)	90.6	96.9	
Physical Activity (Met/h)	1.1 ± 0.13	1.11 ± 0.17	0.733
Serum magnesium (mg/dl)	2.35 ± 0.21	2.25 ± 0.17	0.047

*BMI *Body mass index

^1^Mean ± SD

Table 2 shows intake of nutrients (per 1000 kcal) of subjects during the study. The intake of carbohydrate (*p* = 0.325), protein (*p* = 0.583), fat (*p* = 0.760), cholesterol (*p* = 0.102), linoleic acid (*p* = 0.480), saturated fatty acids (*p* = 0.591), monounsaturated fatty acids (*p* = 0.332), polyunsaturated fatty acids (*p* = 0.959), vitamin A (*p* = 0.434), vitamin E (*p* = 0.704), vitamin K (*p* = 0.403), vitamin C (*p* = 0.086), vitamin B1 (*p* = 0.250), vitamin B2 (*p* = 0.386), vitamin B3 (*p* = 0.532), vitamin B5 (*p* = 0.312), vitamin B6 (*p* = 0.179), folate (*p *= 0.859), magnesium (*p* = 0.481), potassium (*p* = 0.341), calcium (*p* = 0.606), zinc (*p* = 0.560), iron (*p* = 0.609), sodium (*p* = 0.561), and dietary fiber (*p* = 0.412) had no significant differences between the two groups.

**Table 2:** Nutrient intake (per 1000 kcal) of subjects during the study

Nutrients	Magnesium (*n* = 32)	Placebo (*n* = 32)	P
Carbohydrate (g/day)	133.24 ± 102.81	113.11 ± 21.94	0.325
Protein (g/day)	34.04 ± 9.65	32.61 ± 9.16	0.583
Fat (g/day)	48.69 ± 10.85	47.77 ± 11.036	0.760
Cholesterol (mg/day)	111.90 ± 60.55	144.88 ± 81.53	0.102
Linoleic acid (gr/day)	0.32 ± 1.03	0.09 ± 0.14	0.664
Saturated fatty acids (g/day)	10.40 ± 2.56	9.88 ± 4.20	0.591
Monounsaturated fatty acids (g/day)	14.72 ± 11.84	12.35 ± 4.13	0.332
polyunsaturated fatty acids (g/day)	20.45 ± 7.48	20.56 ± 8.33	0.959
Vitamin A (re/day)	287.06 ± 251.52	368.65 ± 466.13	0.434
Vitamin E (mg/day)	1.96 ± 2.041	1.74 ± 2.07	0.704
Vitamin K (ug/day)	36.86 ± 26.47	43.95 ± 34.07	0.403
Vitamin C (mg/day)	68.77 ± 42.92	50.58 ± 32.13	0.086
Vitamin B1 (mg/day)	0.81 ± 0.16	0.75 ± 0.18	0.250
Vitamin B2 (mg/day)	0.84 ± 1.38	0.61 ± 0.12	0.386
Vitamin B3 (mg/day)	11.22 ± 4.03	10.49 ± 4.39	0.532
Vitamin B5 (mg/day)	2.29 ± 0.80	1.98 ± 0.71	0. 312
Vitamin B6 (mg/day)	0.67 ± 0.21	0.74 ± 0.32	0. 179
Vitamin B9 (µg/day)	118.14 ± 48.55	112.70 ± 41.92	0. 859
magnesium (mg/day)	96.79 ± 24.49	91.20 ± 32.12	0.481
Potassium (mg/day)	1035.03 ± 187.17	973.87 ± 267.42	0.341
Calcium (mg/day)	278.53 ± 115.69	265.01 ± 69.31	0.606
Zinc (mg/day)	3.68 ± 1.26	3.38 ± 1.06	0.531
Iron (mg/day)	8.43 ± 2.41	8.80 ± 2.75	0.609
Sodium (mg/day)	517.80 ± 213.30	555.11 ± 248.57	0.561
Dietary Fiber (g/day)	7.12 ± 2.60	6.54 ± 2.52	0.412

^a^ Variables are expressed as mean ± SD

^b^ All variables were adjusted for total energy intake

Table 3 shows the effects of magnesium supplementation on components of quality of life, AUB, alopecia, and acne. In the magnesium group, scores of physical functioning (*p* = 0.011), role limitations due to physical health (*p* = 0.012), role limitations due to emotional problems (*p* < 0.001), energy/fatigue (*p* = 0.005), emotional wellbeing (*p* < 0.001), social functioning (*p* = 0.002), general health (*p* = 0.013), and total quality of life (*p* < 0.001) were significantly improved after intervention compared with baseline. In contrast, number of AUB items (*p* < 0.001) and score of alopecia (*p* = 0.009) decreased after the trial in magnesium group. In placebo group, scores of physical functioning (*p* = 0.028), number of items of AUB (*p* = 0.001) and score of alopecia (*p* = 0.009) were significantly decreased at the end of the trial compared with baseline. More data regarding alopecia, acne, physical activity, AUB scores are presented in the Supplementary File 1.

**Table 3:** The effects of magnesium supplementation on components of quality of life, abnormal uterine bleeding, alopecia and acne ^a^

Variables	Magnesium (*n* = 32)				Placebo (*n* = 32)				P^c^	P^d^
	**Baseline**	**End of trial**	**Change**	**P**^**b**^	**Baseline**	**End of trial**	**Change**	**P**^**b**^		
Components of quality of life										
Score of Physical functioning	60.00 ± 18.66	67.53 ± 18.64	7.53 ± 15.66	0.011*	65.78 ± 26.79	60.31 ± 25.99	-5.46 ± 13.46	0.028*	0.207	0.053
Score of Role limitations due to physical health	38.12 ± 45.66	54.21 ± 46.45	16.09 ± 34.00	0.012*	41.40 ± 48.61	45.46 ± 47.42	4.06 ± 31.09	0.465	0.459	0.093
Score of Role limitations due to emotional problems	9.89 ± 25.34	62.60 ± 42.37	52.70 ± 43.88	< 0.001*	22.92 ± 41.21	29.63 ± 43.09	6.71 ± 29.39	0.206	0.003	0.001*
Score of Energy/ fatigue	30.31 ± 26.42	40.37 ± 24.89	10.06 ± 18.83	0.005*	38.90 ± 28.70	37.18 ± 29.72	-1.71 ± 5.90	0.110	0.644	0.010*
Score of Emotional well being	31.43 ± 25.51	39.84 ± 30.12	14.90 ± 18.26	< 0.001*	42.65 ± 28.05	39.84 ± 30.12	-2.81 ± 14.19	0.271	0.345	< 0.001*
Score of Social functioning	40.00 ± 33.09	54.84 ± 32.04	14.84 ± 25.47	0.002*	49.60 ± 42.65	57.73 ± 53.90	8.12 ± 44.89	0.314	0.795	0.428
Score of Pain	54.29 ± 36.28	60.70 ± 33.40	6.40 ± 19.24	0.069	66.01 ± 32.98	63.59 ± 33.21	-2.42 ± 13.80	0.329	0.730	0.270
Score of General health	43.43 ± 30.25	43.51 ± 30.29	5.20 ± 10.96	0.013*	43.43 ± 30.25	43.51 ± 30.29	0.07 ± 5.33	0.934	0.643	0.042*
Total Score of Quality of life	37.90 ± 15.60	47.26 ± 17.65	9.35 ± 8.67	< 0.001*	47.05 ± 22.26	44.85 ± 21.40	-2.20 ± 7.91	0.126	0.624	< 0.001*
Number of items of AUB	2.13 ± 1.07	1.19 ± 1.03	-0.93 ± 0.94	< 0.001*	1.91 ± 1.32	1.19 ± 1.06	-0.71 ± 1.14	0.001*	0.999	0.651
Score of alopecia	1.94 ± 1.07	1.31 ± 0.93	-0.62 ± 1.26	0.009*	1.94 ± 0.98	1.31 ± 0.93	-0.62 ± 1.26	0.009*	0.999	0.958
Score of acne	1.48 ± 2.791	1.00 ± 2.191	-0.48 ± 1.54	0.092	0.94 ± 2.735	0.13 ± 0.707	-0.81 ± 2.86	0.119	0.041*	0.051

^a^ Variables are expressed as mean ± SD

^b^ Obtained from Paired T test comparing baseline and endpoint values within each group

^c^ Obtained from Independent t-test comparing endpoint measurements between two groups

^d^ Obtained from ANCOVA, adjusted for baseline value of each factor and baseline serum magnesium comparing endpoint values between two groups

^*^P < 0.05

After adjusting for baseline serum magnesium and initial measurements, magnesium supplementation improved scores of role limitations due to emotional problems (*p* = 0.001), energy/fatigue (*p* = 0.010), emotional wellbeing (*p* < 0.001), general health (*p* = 0.042), and total quality of life (*p* < 0.001) compared with placebo.

## Discussion

The results of this study showed that supplementation with 250 mg of magnesium for 10 weeks improved the quality of life components in women with PCOS. Quality of life in PCOS is lower than healthy subjects and those with other gynecological diseases, which can lead to several negative consequences [39, 40, 39]. Therefore, the quality of life in these patients is clinically important [41]. Accordingly, the results of this study suggest that magnesium supplementation might be effective in improving quality of life in PCOS.

Previous studies confirmed that magnesium supplementation had a favorable effect on quality of life. Indeed, a clinical trial showed that oral magnesium sulfate significantly improved the quality of life in women with dysmenorrhea [42], whilst according to another study, it was observed that patients with fibromyalgia had a significant improvement in quality of life by using magnesium supplements [43]. Also, adjuvant therapy with magnesium sulfate reportedly resulted in a significant improvement in quality of life components and beneficial changes in the psycho-emotional state of patients with the chronic coronary syndrome [44]. Moreover, magnesium supplementation improved the quality of life in patients with asthma [45]. Therefore, findings of previous studies regarding improvement of quality of life are concordant with the results of the present study.

We found that magnesium supplementation had no significant effect on acne in patients with PCOS. Acne vulgaris is a cosmetic problem that affects 80% of the population, especially women with PCOS [46]. It is a chronic inflammatory disease with multifactorial causes and clinical manifestations of blackheads, papules, pustules, nodules, and cysts [47]. Using topical magnesium has been reported to result in increased skin hydration and skin permeability, repairing barriers, and facilitating skin proliferation by penetrating beneath the stratum corneum. A local inflammatory process was observed in the skin among subjects with magnesium deficiency [14], and aa cross-sectional study showed that there was a direct association between severity of vulgaris acne and magnesium level [48]; however, the evidence is equivocal. Two clinical trials reported the impact of magnesium containing drugs/supplements on acne. Nevertheless, these studies administered magnesium in combination with other components and drugs. One study used liposomal magnesium in combination with folic acid and topical antibiotic and found that this intervention resulted in improvement of acne[16]. Another study involved 252 adults with acne and used a magnesium-containing medication[49]. Acne severity was improved after using a magnesium-containing drug. It showed that magnesium may have beneficial effects on acne. Since magnesium was not used by itself in these studies, we could not conclude that magnesium was the main cause of acne improvement.

We found that magnesium supplementation had no significant effect on alopecia. We hypothesized that magnesium supplementation may improve alopecia because previous studies showed that magnesium deficiency contributed to alopecia and disrupted cholesterol-enhanced hair loss [50]. Also, topical application of magnesium was reported to be effective in regrowth of shed hair in mice [51]. Nevertheless, a case–control study found that only protein intake was directly effective in alopecia compared to micronutrients including magnesium [52]. Also, a meta-analysis revealed that magnesium deficiency was not a risk factor of hair loss [53].

We did not observe any significant change in AUB after magnesium supplementation. Although some previous studies evaluated the efficacy of magnesium supplementation in PCOS, its effect on AUB was not assessed, or due to publication bias and adverse outcomes, results remain unpublished. Therefore, we could not compare our findings with previous results.

In this study, magnesium supplementation resulted in improvement of physical function and physical health in women with PCOS. According to previous studies, physical activity is associated with increased magnesium requirement and intake [54]. Also, during physical activity, sweating and cell peeling reduce magnesium level [55]. Moreover, there are potential beneficial effects of magnesium supplementation on muscle metabolism and favorable physical function, including improved cardiorespiratory and leg muscle function [56, 57], lower serum total creatine kinase activity, and skeletal muscle creatine kinase isoenzyme [58].

Magnesium supplementation in women with PCOS improved emotional and mental aspects of quality of life. Previous studies showed that low magnesium intake was significantly associated with externalizing behaviors [59], whilst another study found an inverse relationship between dietary magnesium intake and incidence of depression [17]. A review study asserted favorable effects of magnesium supplementation on different types of mental disorder including depressive symptoms, anxiety disorders, attention deficit hyperactivity disorder, autism, obsessive–compulsive disorder, and eating disorders [60]. The antidepressant effect of magnesium is mediated by a variety of mechanisms; indeed, magnesium blocks the N-methyl-D-aspartate glutamatergic receptor, whilst other components of glutamatergic transport, such as the AMPA α-amino-3-hydroxy-5-methyl-4-isoxazole propionic acid receptor, is modified by magnesium [61]. Elevated brain magnesium levels may increase fear memory retention by increasing N‐methyl‐D‐aspartate signaling, brain-derived neurotrophic factor expression, and synaptic plasticity in the body. Therefore, magnesium may increase mental aspects of quality of life by its antidepressant effect.

In the present study, an improvement in the general health of patients with PCOS was observed after magnesium supplementation. Since magnesium is a coenzyme of more than 300 enzymes in the body, and many chemical reactions require sufficient magnesium level, it is unsurprising that the general health of the body depends on adequate intake of magnesium [62]. In clinical practice, optimizing magnesium status through diet and supplements appears to be a safe, useful, and documented treatment for several diseases [63]. Therefore, magnesium supplementation may have a beneficial effect on improving physical, mental, and general health of the body and thus a better quality of life in patients with PCOS. Therefore, according to previous observations and the results of this study, magnesium supplementation can play an effective role in improving the total quality of life.

Several strengths and limitations should be stated with regard to the present study. the Covid-19 pandemic and subsequent lockdown was a reason for several participants ceasing study enrolment. In addition, some variables in the present study were collected by subjective methods, which is often accompanied by recall bias; however, we utilized validated methods in an effort to ameliorate this issue. A strength of the current study was using ITT method in statistical analysis, which was a priori defined. Also, all components of quality of life were reported in the results, allowing detailed insight into numerous aspects of quality of life.

## Conclusion

Magnesium supplementation in women with PCOS had a significant positive effect on improving total quality of life and its components. However, data regarding the effect of magnesium supplementation on alopecia, AUB and acne was not sufficient to draw a consensual conclusion. Future studies should assess the effect of magnesium supplementation on AUB, acne and alopecia.

## Supplementary Information

**Additional file 1.**

## Data Availability

Data will be available on request.

## Declarations

### Transparency Declaration

The lead author affirms that this manuscript is an honest, accurate, and transparent account of the study being reported. The reporting of this work is compliant with CONSORT guidelines. The lead author affirms that no important aspects of the study have been omitted and that any discrepancies from the study as planned. The Research Council and Ethical Committee of Isfahan University of Medical Sciences, Isfahan, Iran and Food Security Research Center, Isfahan University of Medical Sciences, Isfahan, Iran approved this study (Code: IR.MUI.RESEARCH.REC.1399.406). This randomized clinical trial was registered at IRCT.ir on 2020–10-18 (Registration Code: IRCT20130903014551N9).
